# Molecular genetic investigative leads to differentiate monozygotic twins

**DOI:** 10.1186/2041-2223-5-11

**Published:** 2014-08-29

**Authors:** Bruce Budowle

**Affiliations:** 1 Department of Molecular and Medical Genetics, Institute of AppliedGenetics, University of North Texas Health Science Center, Fort Worth, Texas, USA; 2 Center of Excellence in Genomic Medicine Research (CEGMR), King Abdulaziz University, Jeddah, Saudi Arabia

## 

Monozygotic twins arise from a single fertilized egg and, thus, for practical purposes are genetically identical. Therefore, in cases where one of the identical twins is associated with forensic biological evidence through DNA typing, the other twin cannot be excluded either. This conundrum of the ultimate “my brother did it” scenario has at times placed the legal community in a difficult position for solving the source of biological evidence in such cases. Indeed, identical twins are expected to have the same, for example, short tandem repeat loci, profile, no matter how highly individualizing the current forensic DNA diagnostic system is.

Although the dogma is that monozygotic twins are genetically identical, it has been well-known for many years that there are genetic differences between such twins due to the accumulation of somatic mutations [1-8]. Undeniably, a few somatic DNA differences between twins are the norm, not the exception. The earlier in the embryonic development of an individual that a somatic mutation occurs, the more prevalent it will be among the tissues of that particular individual. Somatic mutations occur randomly, so it is extremely unlikely, if not impossible, that identical twins would share the same somatic mutations. These mutations, thus, potentially can serve as genetic markers to distinguish identical twins and thus resolve the dilemma of which one is likely to be the source of the biological evidence, if such a circumstance arises.

Until recently, finding these few somatic mutations was not feasible routinely and very demanding technically. Over the last decade the advent of massively parallel sequencing (MPS) makes possible grand scale sequencing in which whole human genomes can be sequenced in a relatively rapid time frame and has ushered in a genetics revolution. Even forensic DNA analyses are being driven in this new direction with the promising capabilities provided by MPS. While much effort is being dedicated to the application of MPS in forensic DNA analysis, it is still considered by most in the development and validation testing phases and is not being used in casework analyses. However, recently Weber-Lehmann et al. published “Finding the needle in the haystack: Differentiating ’identical’ twins in paternity testing and forensics by ultra-deep next generation sequencing” [9] in which they exploit the power of MPS and show that the technology may assist even today in casework where pinpointing genetic differences between twins is desired.

The authors sequenced reference DNA with the Illumina HiSeq 2000 platform using chemistry v3.0 and 2 × 100 bp paired-end read mode from sperm samples of two identical twins (monozygosity confirmed using PowerPlex 21 PCR Kit; Promega, Madison, WI) and from a blood sample of the child of one twin. With such high throughput of approximately 600 gigabases, whole genome sequence data were generated resulting in about 90X coverage on average per twin. The child’s genome was sequenced on average to 56X. After bioinformatics processing to remove noisy data, five single nucleotide polymorphisms (SNPs) were observed in the father and child pair that were not detected in the other twin (i.e., the child’s uncle). Once these SNPs were identified, directed PCR assays were readily developed and standard Sanger sequencing verified the SNPs. Thus, the validity of the SNPs was bolstered by orthogonal testing. The mother of the child was tested for these SNPs using the directed assay, and she was excluded as the source of the child’s SNPs.

This study demonstrated that it is possible and feasible to identify somatic differences between twins. Four of the five SNPs that the twin father carried were in both his buccal and sperm DNA (ectodermal tissues). One of the SNPs was detected in both sperm and blood DNA. Lastly, one SNP was observed solely in sperm DNA. These findings support the hypothesis that the earlier in development of an embryo, rare SNPs may arise and be established not only in somatic tissues, but importantly in the germline.

The implication of these findings for forensic investigations involving twins as the potential source is remarkable. Suppose there is a sexual assault case, semen is discovered, typed by standard DNA markers, and a “match” is observed between the evidence and an implicated twin. The primary question of differentiation of the twin who is the donor of the semen evidence can be resolved. Ideally, sequencing semen reference samples would be better for making comparisons by MPS; yet, obtaining such reference samples is unlikely. However, as long as the relevant somatic mutations that will be identified in a buccal or blood reference sample by MPS also reside in sperm DNA, the ability to differentiate the twins is highly probable. Using this investigative approach, the risk of false identification is infinitesimally low with the likely outcomes being differentiation or inconclusive. Indeed, only one or two SNPs would be needed to distinguish between the twins and there does not appear to be a need for additional statistical analyses of the outcome. Traditional STR typing already would have reduced the possible source of the biological evidence to be most likely that of one of the twins as opposed to other potential contributors.

It is important to note that MPS is not being used to analyze the semen evidence with this approach. MPS is being used solely on reference samples to develop the investigative lead for identifying target SNPs. Once genetic variants are identified, typing of the evidence and the reference samples of the identical twins would be carried out using similar methodology which has been generally accepted for sequencing of mitochondrial DNA for almost two decades, i.e., targeted PCR and Sanger sequencing. Thus, the methodology that would be used for forensic analysis of the semen evidence and reference samples would be well-established and generally-accepted. Typing the evidence, and again the references samples, to detect those genetic variants using standard sequencing technology is an additional feature for supporting the reliability of the resultant SNPs. Lastly, using this approach, there is far less consumption of precious evidence than would be if MPS were used directly on the sperm DNA to identify SNPs. So, there will be, in at least some cases, sufficient DNA for retesting, if it were desired.

Weber-Lehmann et al. [9] have shown that there currently are other practical uses of MPS for forensic investigations. The leads are those precious few markers that can differentiate monozygotic twins. While the authors use the catchy phrase “finding the needle in the haystack,” they have shown in actuality that it is not so difficult. But for now it likely will not be a cheap investigation as the genomes of each twin pair will have to be sequenced to identify the novel somatic SNPs that would differentiate them.

## Competing interests

The author declares that he has no competing interests.
